# Added value of magnetic resonance spectroscopy for diagnosing childhood cerebellar tumours

**DOI:** 10.1002/nbm.4630

**Published:** 2021-10-13

**Authors:** Nigel P. Davies, Heather E. L. Rose, Karen A. Manias, Kal Natarajan, Laurence J. Abernethy, Adam Oates, Umair Janjua, Paul Davies, Lesley MacPherson, Theodoros N. Arvanitis, Andrew C. Peet

**Affiliations:** ^1^ Institute of Cancer and Genomic Sciences University of Birmingham Birmingham UK; ^2^ Department of Medical Physics University Hospitals Birmingham NHS Foundation Trust Birmingham UK; ^3^ Birmingham Women's and Children's Hospital NHS Foundation Trust Birmingham UK; ^4^ Alder Hey Children's Hospital NHS Foundation Trust Liverpool UK; ^5^ Institute of Digital Healthcare, WMG University of Warwick Coventry UK

**Keywords:** ^1^H‐magnetic resonance spectroscopy, brain tumours, classification, metabolites, paediatric

## Abstract

^1^H‐magnetic resonance spectroscopy (MRS) provides noninvasive metabolite profiles with the potential to aid the diagnosis of brain tumours. Prospective studies of diagnostic accuracy and comparisons with conventional MRI are lacking. The aim of the current study was to evaluate, prospectively, the diagnostic accuracy of a previously established classifier for diagnosing the three major childhood cerebellar tumours, and to determine added value compared with standard reporting of conventional imaging. Single‐voxel MRS (1.5 T, PRESS, TE 30 ms, TR 1500 ms, spectral resolution 1 Hz/point) was acquired prospectively on 39 consecutive cerebellar tumours with histopathological diagnoses of pilocytic astrocytoma, ependymoma or medulloblastoma. Spectra were analysed with LCModel and predefined quality control criteria were applied, leaving 33 cases in the analysis. The MRS diagnostic classifier was applied to this dataset. A retrospective analysis was subsequently undertaken by three radiologists, blind to histopathological diagnosis, to determine the change in diagnostic certainty when sequentially viewing conventional imaging, MRS and a decision support tool, based on the classifier. The overall classifier accuracy, evaluated prospectively, was 91%. Incorrectly classified cases, two anaplastic ependymomas, and a rare histological variant of medulloblastoma, were not well represented in the original training set. On retrospective review of conventional MRI, MRS and the classifier result, all radiologists showed a significant increase (Wilcoxon signed rank test, *p* < 0.001) in their certainty of the correct diagnosis, between viewing the conventional imaging and MRS with the decision support system. It was concluded that MRS can aid the noninvasive diagnosis of posterior fossa tumours in children, and that a decision support classifier helps in MRS interpretation.

## Graphical abstract

High diagnostic accuracy was achieved in the prospective evaluation of a ^1^H‐magnetic resonance spectroscopy (MRS) diagnostic classifier previously developed for childhood cerebellar tumours. A radiologist's diagnostic accuracy was significantly improved when standard conventional imaging was supplemented by a clinical decision support system including the MRS classifier.

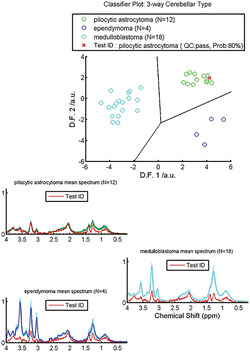

**Abbreviations used**
Ala: alanine
Cr: creatine
Glc: glucose
Gln: glutamine
Glu: glutamate
GPC: glycerophosphocholine
Gua: guanidinoacetate
Lac: lactate
Ins: *myo*‐inositol
NAA: N‐acetylaspartate
PCh: phosphocholine
Scyllo: *scyllo*‐inositol

## 1 INTRODUCTION

Brain tumours are the leading cause of cancer deaths in children,^1^ with a range of treatment options and outcomes depending on the tumour type, its location and the patient’s age.^2^ Histopathology, following biopsy or resection, is the current gold standard for diagnosis of brain tumour type and grade.^2^ As a definitive histopathological diagnosis is not available until several days postoperatively, this cannot be used to guide surgical decision‐making or early planning of adjuvant treatments, such as chemotherapy or radiotherapy. While this delay does not have a definitive impact on survival,^3^ prompt and accurate discussions with the family relating to diagnosis and treatment planning can provide reassurance at a difficult time.^4^ Radiological diagnosis of children's brain tumours is routinely made by qualitative interpretation of conventional magnetic resonance images, such as structural T_1_‐ and T_2_‐weighted scans.^5^, ^6^ However, focus is shifting to the diagnostic value of advanced, quantitative, MR techniques that provide additional information on tissue properties, such as diffusion‐weighted imaging, perfusion imaging and magnetic resonance spectroscopy (MRS).^7^, ^8^, ^9^, ^10^

^1^H‐MRS provides noninvasive measurement of metabolite profiles,^11^ with the potential to aid diagnosis and improve the characterisation of brain tumours.^12^, ^13^, ^14^, ^15^, ^16^ Coupling metabolite profiles with machine learning techniques furthers diagnostic classification potential.^9^, ^15^, ^17^ Diagnostic classifiers, based on MRS, for brain tumours in adults, have been evaluated both retrospectively and prospectively,^18^, ^19^, ^20^, ^21^ showing good accuracy for discriminating between certain common tumour types. Some studies go further, aiming to correlate MRS profiles and the genetic profiles of adult gliomas.^22^ Specific classifiers are required for childhood brain tumours, as the common paediatric tumour types differ from those occurring in adults.^2^ Both single and multicentre studies of MRS for classifying childhood brain tumours have been reported for the three main tumour types: pilocytic astrocytoma (PA), medulloblastoma (MB) and ependymoma (EP).^17^, ^19^ These studies have, however, been retrospective, and there has been little systematic comparison with conventional radiological reporting of MRI.

Studies adding visual interpretation of MRS profiles to conventional MRI have been found to significantly improve radiologists' diagnostic accuracy of paediatric brain tumours.^23^, ^24^, ^25^ Interpretation of MRS in addition to MRI, by a radiologist, was found to provide clinical benefit in selective cases. There is also some evidence that qualitative interpretation of MRS improves the accuracy of noninvasive diagnosis in the clinical environment.^26^ However, while the highest accuracies of diagnosis from MRS for children's brain tumours have been achieved using quantitative analysis in combination with machine learning, these classifiers have not been prospectively evaluated, nor has the added value of this approach over radiological reporting of conventional MRI been systematically studied in children's brain tumours. Ongoing methodological improvements in the development of machine learning classifiers discourages prospective evaluation because the classifiers evaluated will not use current optimal methods; however, such studies are a key step in providing the evidence required for clinical adoption.

The aim of this study was twofold: firstly, to take a previously reported MRS classifier for discriminating between the three main childhood cerebellar tumours^15^ and perform a robust prospective evaluation, without classifier adaptation; and secondly, to assess the value of MRS and the classifier output, when added to conventional radiological reporting.

## 2 METHODS

### 2.1 Patients

Ethical approval was granted by the NHS Research Ethics Committee and parental informed consent was obtained. Patients imaged using MRS, prior to treatment for a brain tumour located in the posterior fossa, during 1 November 2006–31 October 2010, and subsequently diagnosed by histopathology, were eligible for participation in this prospective study.

### 2.2 Data acquisition

All studies were performed using one of two 1.5‐T scanners (Siemens Symphony Magnetom, NUM4 SQ‐Engine Gradients, 45 mT/m, SR 200, software version: Syngo MR 2004a, 16‐channel head coil; and GE Signa Excite Hd/x.33/120 EchoSpeed Plus Gradients, software version: 15.0, eight‐channel head coil). The standard imaging protocol consisted of T_1_‐, T_2_‐ and diffusion‐weighted images of the brain, and T_1_‐weighted images of the head and spine, following contrast agent administration. MRS was acquired using a point‐resolved single voxel spectroscopy (PRESS) sequence (TE 30 ms, TR 1500 ms, 1024 or 2048 complex points, filter bandwidth 2000–2500 Hz) with an acquisition bandwidth per point of 1.024 Hz. Cubic voxels of 2‐ or 1.5‐cm length were used with 128 or 256 repetitions, respectively. Scanner CHESS sequences were used for water suppression; no out of volume suppression was used. Water reference spectra, with eight repetitions and all other acquisition parameters the same as the corresponding water‐suppressed sequences, were acquired for eddy current correction and as an internal reference for quantifying metabolite concentrations. Conventional images were used to delineate the margins of the primary tumour and enable placement of the MRS voxel within the solid‐appearing component of the tumour, avoiding areas of cyst or normal appearing brain tissue. The risk of lipid contamination of the MRS signal, from scalp or other fatty tissue, was minimised by avoiding close proximity to these areas when positioning the voxel.

### 2.3 MRS processing

**FIGURE 1:**
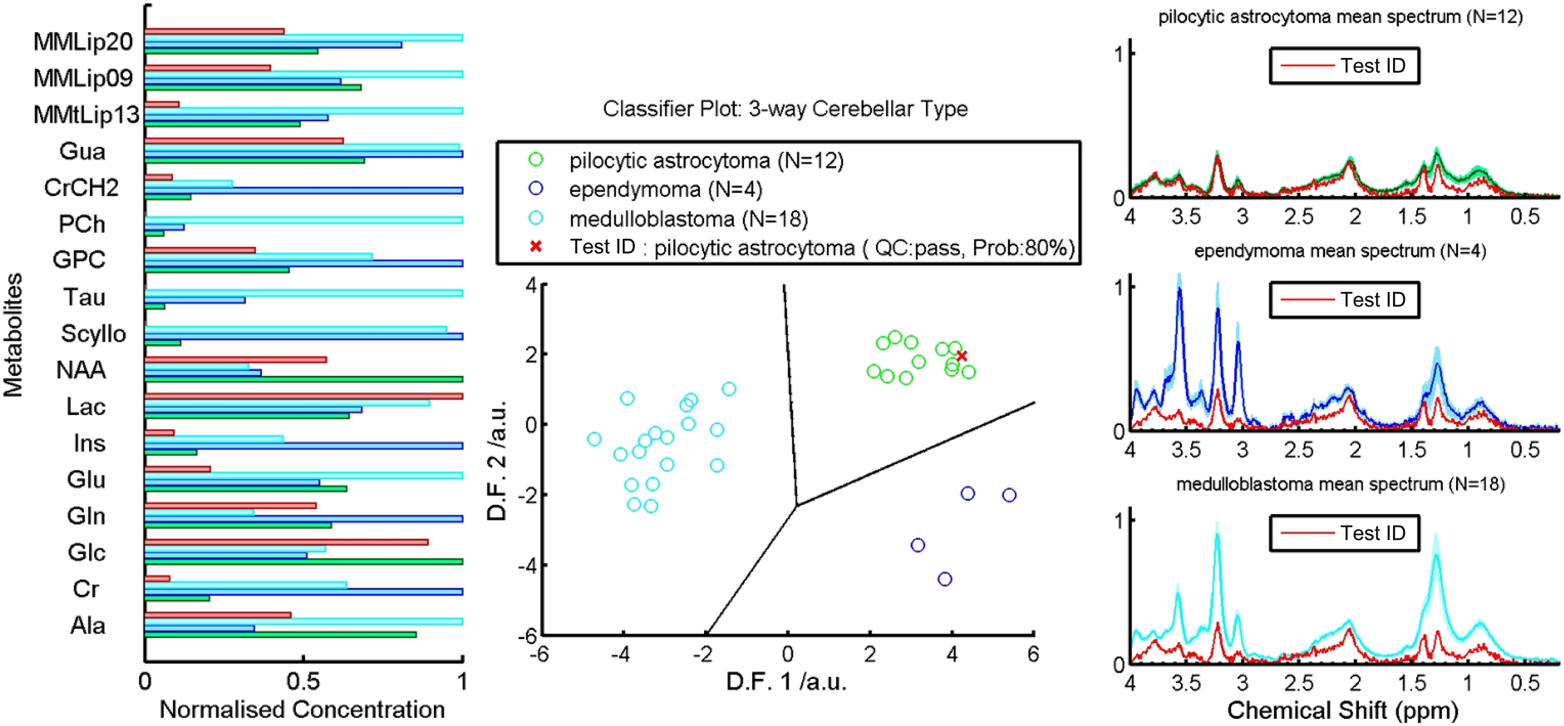
Example of the DSS output presented to the radiologists showing the normalised metabolite profiles (left), linear discriminant function (D.F.) scores (centre) and MR spectra for the index case (red) compared with the mean values for each tumour type (green = pilocytic astrocytoma, blue = ependymoma and cyan = medulloblastoma) (right) in the training dataset

MRS data were processed and fitted using the LCModel (version 6.1–4) software package^27^ using the integrated fitting designed for spectra with weaker N‐acetylaspartate (NAA) signals, SPTYPE = Tumor and its associated default setting, as defined in the LCModel manual. DELTA and NULFIL were extracted from the scan file header and the H2O suppression flag set to TRUE. All other parameters were maintained at default, including DKNTMN at 0.15 ppm. Postprocessing included zero‐ and first‐order automatic phasing, and eddy current correction based on water reference spectra. The basis set contained spectra from 16 different metabolites and nine simulated lipid and macromolecular components, with a simulated negative singlet at 3.94 ppm (‐CrCH2), as described in the LCModel manual. Estimated concentrations were obtained, using the spectrum acquired without water suppression as a reference, with an assumed water concentration of 35,880 mM. The macromolecular and lipid components were grouped together to fit resonances at approximately 0.9 ppm (MMLip09), 1.3 ppm (MMLip13) and 2.0 ppm (MMLip20), giving 19 variables in all. Metabolites with a Cramer–Rao lower bound of less than 30%, in at least two subjects, were included in the analysis. The same quality control (QC) criteria were applied to the test cohort as were applied to the training cohort, as previously described.^15^ The LCModel baseline for each spectrum was subtracted, and the spectra normalised to the corresponding unsuppressed water peak, prior to generating mean spectra for each patient group. This method of normalisation assumes relatively stable water content within a tumour group but allows ease of comparison with the literature provided to participating radiologists. LCModel‐estimated concentrations were used for the machine learning‐based classification, LCModel spectra for the training cohort were used to generate mean MRS spectra for each tumour type and the index spectra for each case, displayed in the MRS decision support system (DSS) output (Figure 1). MRS processed using scanner software was also prepared for evaluation as a noninteractive image screen capture. For the Siemens Symphony Magnetom Num 4, the baseline was corrected using subtraction of a polynomial fitted baseline. The spectral range was 0.5–4.30 ppm with automatic phase correction, and a Hanning filter (width 700 ms) and zero filling were applied. Lorentzian curve fitting was undertaken and displayed for NAA, creatine (Cr), choline and *myo*‐inositol (Ins), including the calculated peak integrals. For scanner processing on the GE Signa Excite Hd/x, the spectra range was 0–4.30 ppm; no processing was carried out beyond automatic phasing and Fourier transform to produce the frequency domain spectra.

### 2.4 Classification and confidence measure

Classification accuracy was assessed using the previously published classifier for PA, EP and MB.^15^ Each individual test case was classified using the data processing pathway, defined by the previously optimised classifier, without any alterations. After standardising the metabolite values according to the mean and standard deviation of the training dataset, each case was classified by applying the linear discriminant function coefficients derived from the classifier described in previous work.^15^ The accuracy of the classifier is taken as the percentage of the total number of tumours with a correct diagnosis. The posterior probability for each tumour was given as a measure of the confidence, which could be placed in the diagnosis given by the classifier.

### 2.5 Conventional MRI reporting and added value of MRS

A study of the diagnostic accuracy of standard radiologist reporting of conventional imaging, followed by the added value of MRS and a DSS based on the MRS classifier, was undertaken. Conventional imaging (MRI and CT, where available) was anonymised and reported retrospectively by three paediatric radiologists. One radiologist was a consultant, with more than 13 years of experience working in the neuro‐oncology multidisciplinary team and was involved in local and national children's brain tumour research. The second was a consultant with less experience (4 years), but who was also a member of the local neuro‐oncology multidisciplinary team. The third was a senior trainee radiologist on a paediatric placement. None of the radiologists had been involved in reporting the patients' MRI or CT at the time of diagnosis. All reporting was undertaken blind to histopathological diagnosis and independently from the other two radiologists. Only cases with MRS that passed QC were included.

Radiologists were given a letter with instructions and a worked example prior to undertaking reporting, in addition to recommended literature to review.^15^, ^28^, ^29^ They were told that all cases had a diagnosis of either PA, MB or EP, and were given the age and gender of the child. Radiologists were asked to give their subjective probability estimate (subsequently referred to as ‘certainty’) of each diagnosis (as a percentage, with the three adding up to 100%) after viewing sequentially (1) conventional imaging, (2) MRS‐processed using scanner software, and (3) the MRS DSS output (Figure 1).

### 2.6 Statistical analysis

Mean metabolite concentrations were calculated and compared between the tumour types and the training and testing cohorts using one‐way ANOVA and multiple comparison Student's T‐tests.^15^ Diagnostic accuracy for the radiologist review was quantified as the percentage of correct diagnoses made with a certainty of more than 50%. Mean diagnostic accuracy and the certainty assigned to each histological diagnosis were compared at each review stage for each radiologist. The added value of MRS was assessed by analysing the sequential change in certainty assigned to the correct histological diagnosis, firstly after visual inspection of the MRS, and secondly after viewing the DSS output. For each assessor, the changes in certainty, assigned to the correct histological diagnosis from those made at the previous stage for each case, were calculated and the group median changes assessed, using the Wilcoxon signed rank test. The distribution of the subjective certainty values made the use of procedures based on the normal distribution inappropriate.

## 3 RESULTS

### 3.1 Patient cohort

Details of the original cohort for training the classifier^15^ and the cohort for the prospective study are shown in Table 1. There were no significant differences in age or gender between the cohorts, although there was a higher proportion of PA relative to MB in the test cohort.

**TABLE 1:** Case mix comparison for the training and prospective testing patient cohorts

	Tumour type	Demographics									
		Training set					Test set				
		N	M/F	Age/y			N	M/F	Age/y		
				Range	Mean	SD			Range	Mean	SD
**Before QC**	**Pilocytic astrocytoma**	13	7/6	3.3–15.5	9.0	3.9	21	9/12	2.1–14.6	8.9	4.1
	**Ependymoma**	5	3/2	1.0–3.3	1.9	1.1	4	3/1	2.4–16.3	6.0	6.8
	**Medulloblastoma**	18	12/6	2.4–15.1	7.0	3.9	14	12/2	2.1–13.8	7.9	3.8
	**All**	36	22/14	1.0–15.5	7.2	4.3	39	24/15	2.1–16.3	8.2	4.3
**After QC**	**Pilocytic astrocytoma**	12	6/6	3.3–13.2	8.4	3.3	16	8/8	2.1–13.7	9.3	4
	**Ependymoma**	4	2/2	1.0–3.3	2.0	1.1	3	2/1	2.4–2.8	2.6	0.2
	**Medulloblastoma**	18	12/6	2.4–15.1	7.0	3.9	14	12/2	2.1–13.8	7.9	33.8
	**All**	34	20/14	1.0–15.1	7.1	4.1	33	22/11	2.1–13.8	8.1	4.1

Abbreviation: QC, quality control.

### 3.2 Classifier

**FIGURE 2:**
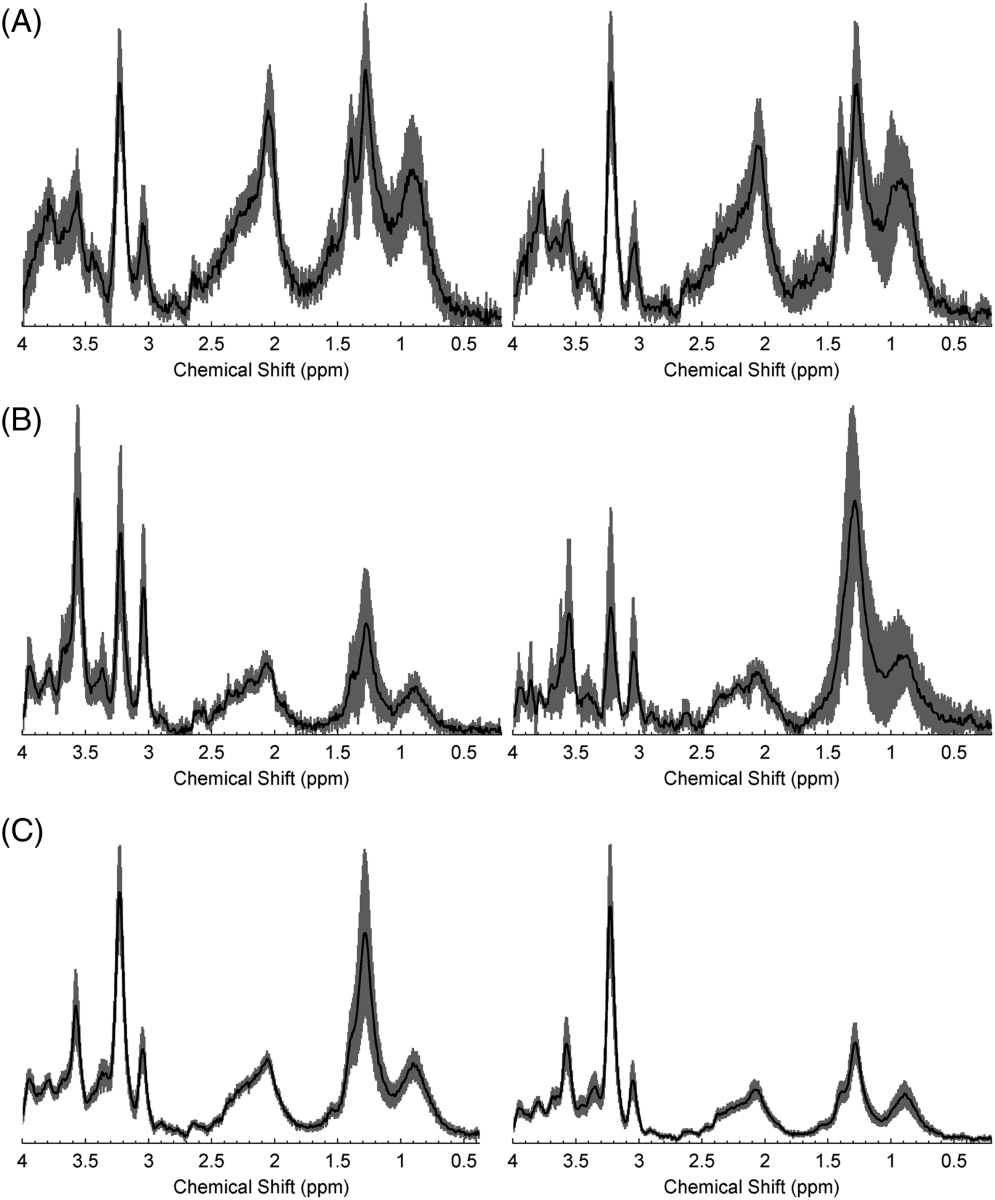
Comparison of mean spectra from the training (left) and prospective testing (right) cohorts for (A) pilocytic astrocytomas, (B) ependymomas and (C) medulloblastomas, with 95% confidence intervals indicated by the shaded region

**FIGURE 3:**
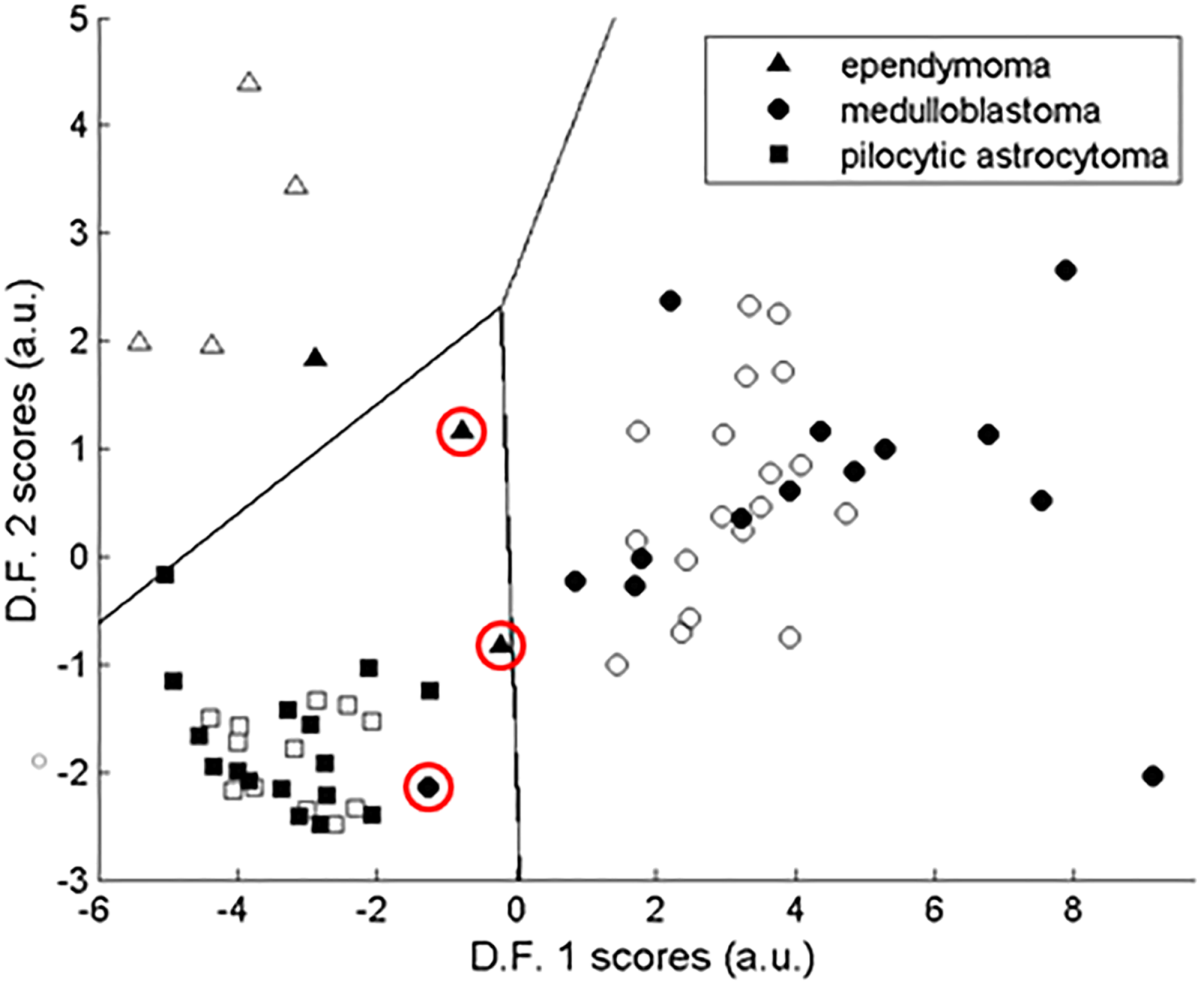
Scatter plots showing the linear discriminant analysis (LDA) scores and decision boundaries for the three‐class classifier of paediatric cerebellar tumours with the training and test set scores represented by open and solid‐filled symbols, respectively. Incorrectly classified cases are indicated by red circles

Figure 2 shows the mean spectra for each tumour type in the training and testing cohorts, revealing some differences in individual peaks but broadly similar patterns. Figure 3 illustrates the prospective classifier results, showing plots of the discriminant function (DF) scores. The DF scores for the training cohort are also plotted to show the extent of overlap between the two cohorts. The distance of the DF scores, for each test case, from the decision boundaries between tumour types reflects the certainty of the classification.

**FIGURE 4:**
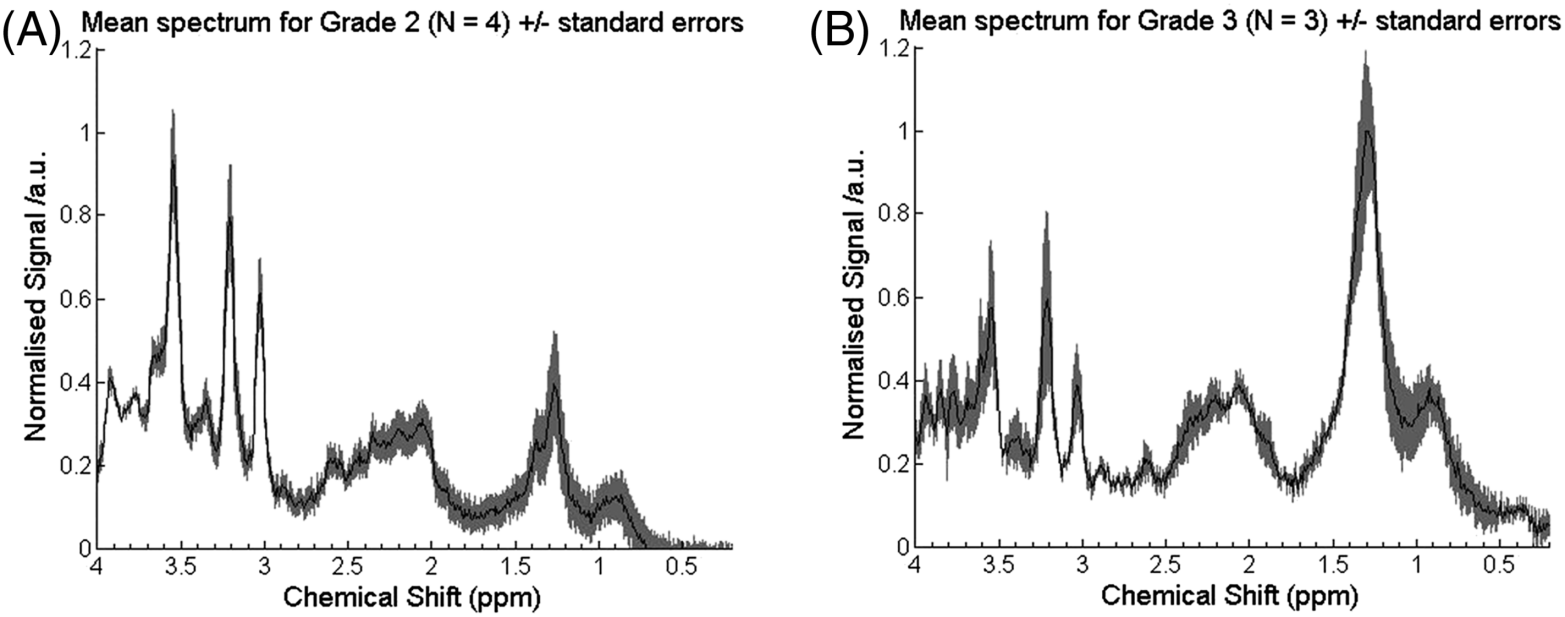
Comparison of mean spectra for (A) grade 2 and (B) grade 3 ependymomas

The overall classification accuracy was 91%, with three out of 33 cases being classified incorrectly. One patient with MB was misclassified prospectively as PA. This case was a rare histological variant with myoblastic elements, generally considered to have poor prognosis: this patient died following tumour progression. Two patients with grade 3 EP were misclassified as PA. Their MRS lay close to the boundary between tumours and had a posterior probability of less than 50%. A comparison of mean spectra, for grade 3 (N = 3) and grade 2 (N = 4) EPs, is shown in Figure 4, with grade 3 tumours having higher lipids (*p* < 0.05, Kruskal–Wallis test).

### 3.3 Radiological review

Table 2 shows the diagnostic accuracy and the mean certainties for the correctly and incorrectly diagnosed cases. It should be noted that while a certainty of more than 50% was required to be assigned to the correct histological diagnosis for diagnostic accuracy purposes, the ‘certainty when correct’ values include cases where the highest certainty was assigned to the correct diagnosis, even if it was 50% or less. For all three radiologists, there was a progressive increase in diagnostic accuracy from standard imaging to standard imaging plus MRS, to standard imaging plus MRS and the DSS. Diagnostic certainty was also increased by viewing the MRS and more so by using the DSS when the diagnosis was correct. All radiologists had the same overall accuracy of diagnosis, after viewing the DSS output.

**TABLE 2:** Diagnostic accuracy with number of correct diagnoses in brackets for each radiologist and their reported certainty in the diagnosis when correct and not correct compared (with standard deviation in brackets) with histopathology using standard imaging (MRI + CT where available), and at each stage after viewing the MRS spectrum produced by the scanner software and subsequently the DSS output

		Data used for radiology review		
		Standard imaging	Standard imaging + MRS	Standard imaging + MRS + DSS
Diagnostic accuracy	Rad 1	73% (N = 24)	82% (N = 27)	88% (N = 29)
	Rad 2	76% (N = 25)	82% (N = 27)	88% (N = 29)
	Rad 3	42% (N = 14)	61% (N = 20)	88% (N = 29)
Mean (SD) certainty when correct	Rad 1	78% (14%)	88% (14%)	89% (14%)
	Rad 2	76% (15%)	79% (14%)	86% (10%)
	Rad 3	56% (8%)	62% (11%)	71% (11%)
Mean (SD) certainty when incorrect	Rad 1	72% (22%)	58% (16%)	63% (5%)
	Rad 2	69% (21%)	69% (18%)	68% (10%)
	Rad 3	44% (8%)	40% (0%)	50% (0%)

**FIGURE 5:**
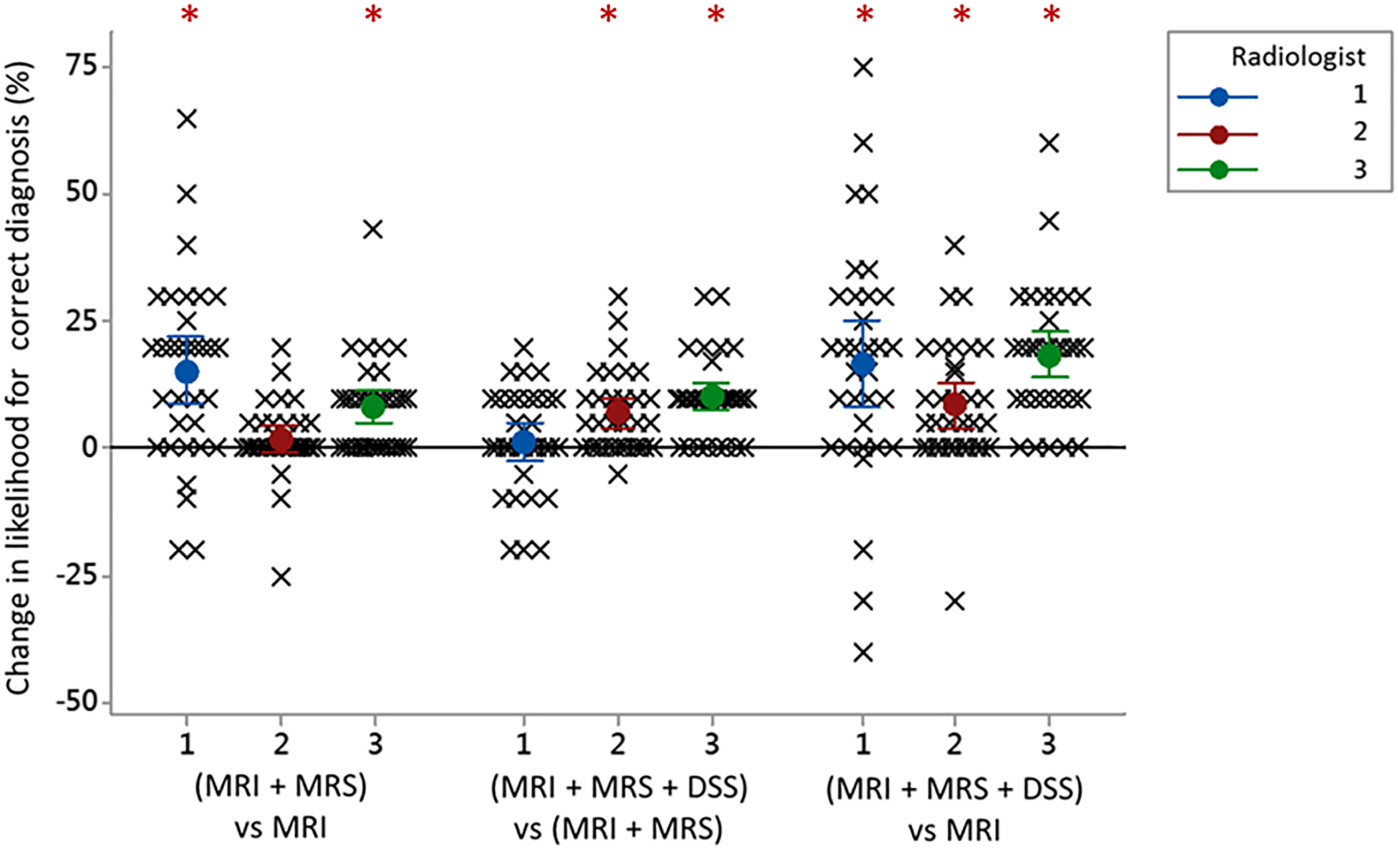
Scatter plot demonstrating the added value of MRS for noninvasive diagnosis of childhood posterior fossa tumours compared with conventional imaging alone. The change in certainty (likelihood) assigned to the correct diagnosis by each of the radiologists (labelled 1, 2 and 3) is shown for each case after qualitative assessment of the MRS (MRI + MRS) and the DSS output (MRI + MRS + DSS). Significant increases in the certainty of correct diagnosis are indicated by * (Wilcoxon signed rank test, *p* < 0.001). The mean with 95% confidence intervals is also shown for each radiologist at each stage

Figure 5 details the change in certainty assigned to the correct histological diagnosis by each radiologist for each case, after using the MRS and DSS, compared with standard imaging alone. This includes certainty changes where the correct histological diagnosis was initially ranked second or third by the radiologists but was then identified as the correct diagnosis when MRS or MRS and DSS data were provided. All three radiologists showed a significant increase (Wilcoxon signed rank test, *p* < 0.001) in their certainty of the correct diagnosis between initial and final stages. The three median increases and their 95% confidence intervals were 16.5 (10.0–25.0), 7.5 (5.0–12.5) and 20.0 (15–22.5). For radiologists 1 and 3, significant increases in certainty of the correct diagnosis were found after purely viewing the MR spectra compared with conventional imaging alone (Wilcoxon signed rank test, *p* < 0.001). Radiologist 3 showed a further significant increase in certainty of the correct diagnosis after viewing the DSS (Wilcoxon signed rank test, *p* < 0.001), while for radiologist 2 the significant increase only occurred after viewing the DSS results (Wilcoxon signed rank test, *p* < 0.001). Levene's test showed significant differences (*p* < 0.01) in the variability of the radiologists' increases with standard deviations of 23.6, 12.5 and 13.2, respectively. There was no relationship between the median increase in the radiologist's certainty of the correct diagnosis, or its variability, and their level of experience. While there are not enough cases for a robust statistical analysis, there were no obvious differences in diagnostic accuracy between tumour types for the radiologists.

**FIGURE 6:**
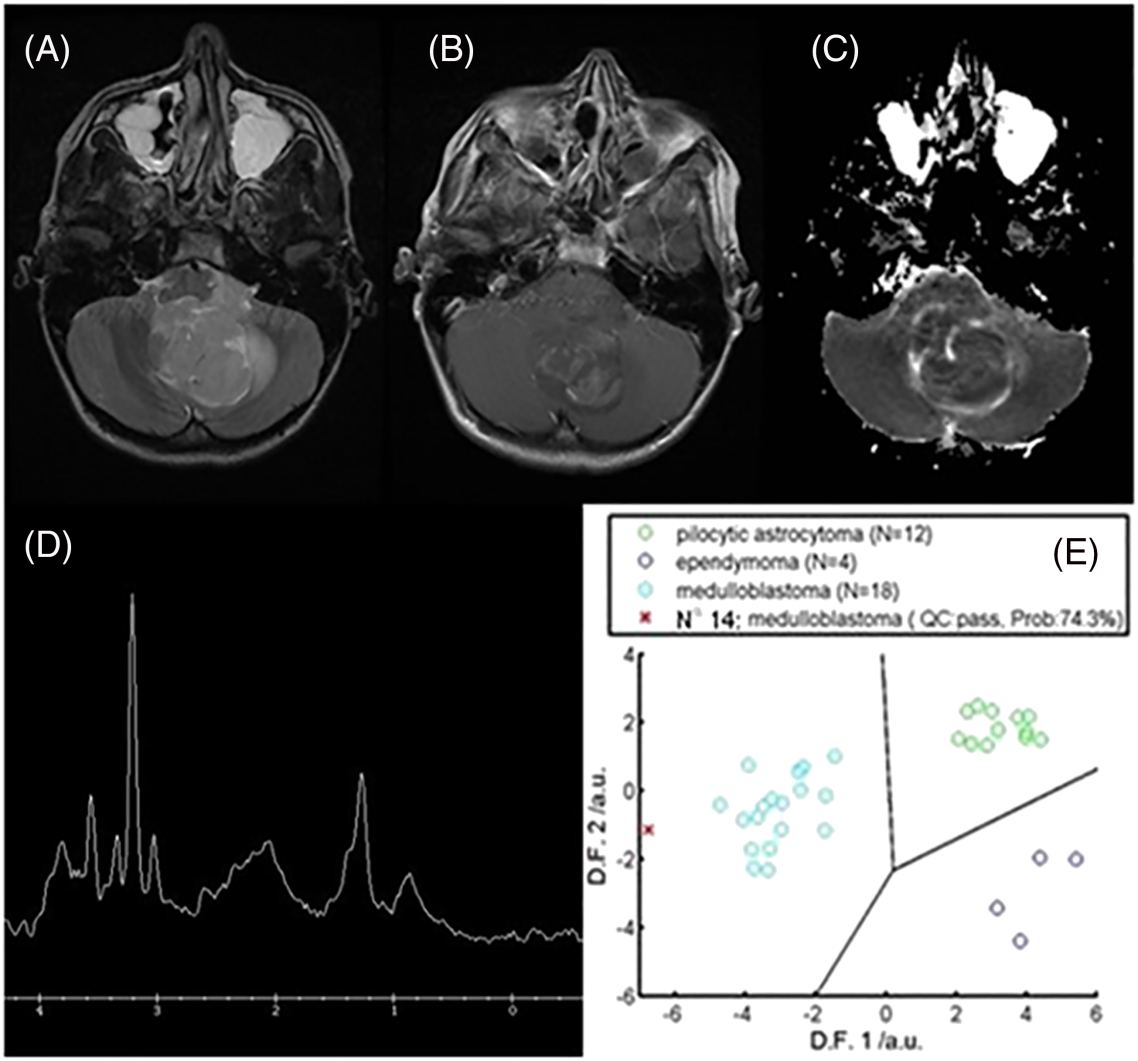
A 13‐year‐old boy presented with headaches and vomiting and was found to have a 4.2 x 3.6 x 2.4 cm heterogeneous mass lesion in the right cerebellar hemisphere on MRI. The lesion contained multiple cystic areas and prominent vessels and demonstrated restricted diffusion. MRS demonstrated a high Cho peak, low Ins and the presence of taurine, a pattern observed in medulloblastoma. Histology confirmed a diagnosis of medulloblastoma 8 days after surgical resection. The figure shows (A) T2‐weighted MRI, (B) postcontrast T1‐weighted MRI, (C) an apparent diffusion coefficient (ADC) map, (D) MRS processed by scanner and (E) DSS output. The radiologists' assigned certainty for the correct diagnosis increased from 50% to 80%, from 45% to 75%, and from 40% to 60% for radiologist 1, 2 and 3, respectively, with the inclusion of MRS and DSS

An example case is presented in Figure 6. Using conventional imaging alone the radiologists ascribed probabilities of 5%, 50% and 20% to the diagnosis of MB. Using MRI alone, radiologists 1 and 3 misdiagnosed this case as PA, with certainties of 90% and 60%, respectively. Incorporation of MRS information resulted in diagnostic probabilities of MB of 70%, 65% and 70%, and review of the DSS output resulted in a further improvement to 80% certainty for all three radiologists. Following surgical resection confirming MB, the child was treated with craniospinal radiotherapy and chemotherapy and made a good recovery.

## 4 DISCUSSION

This study prospectively evaluated a previously published diagnostic classifier, based on MRS, for the three major cerebellar tumours in children and assessed its potential to add value to conventional radiological reporting. A diagnostic accuracy of 91% was found for discriminating between PA, EP and MB, in keeping with results from our previous retrospective study.^15^ High classification rates are seen despite the relatively small number of cases used to train the classifier and the inclusion of data from more than one scanner type. When added to conventional imaging, visual interpretation of MRS and the use of a decision support tool sequentially improved the accuracy and certainty of a radiologist's diagnosis.

Mean MRS for tumours of the three tumour types, acquired prospectively, were very similar to those reported previously (Figure 2). The most notable differences were in EPs, where the lipid peaks were larger, consistent with a more aggressive nature,^30^ and reflect the composition of the datasets. Differences between training and test sets are an important reason for misclassification, as reflected by two of the three misclassified tumours being grade 3 EPs, which were not well represented in the training set. Interestingly, in the original study, the one grade 3 EP in the training set was misclassified, indicating that the classifier did not accurately reflect this tumour group. Improved classifier accuracy should be obtained by the inclusion of more cases, most likely from multicentre data. Alternatively, it has been reported that improved accuracy can be achieved by artificially synthesising extra EP cases for the training set.^9^

It is now largely accepted that MBs form four molecular genetic subtypes and there is evidence that the MRS profiles differ between the subtypes.^31^ Despite this heterogeneity, the MRS profiles of MBs have sufficient similarities to give high classification accuracies. One MB was misclassified, by the three‐way classifier, as a PA. This tumour was particularly interesting, because it was a rare myoblastic variant with distinct histopathological features and an aggressive clinical course. As a general principle, misclassified tumours are likely to have features atypical of common variants well represented in the training set. This may give valuable information for clinical management, as these tumours may not respond to conventional treatment.

One PA, although classified correctly, fell very close to the boundary with EP (Figure 3). MRS of this case revealed a higher amount of Cr than generally seen in PAs, more in keeping with EP. Subsequent review of the images showed the voxel contained a small amount of normal appearing brain, known to contain high levels of Cr, thus accounting for the MRS appearances.^32^ This case demonstrates the importance of voxel placement, as part of QC, when acquiring and interpreting MRS data. It also, however, indicates the technique has some robustness to such problems.

It is encouraging that visual inspection of MRS and the DSS improve both the accuracy of diagnosis and the certainty assigned to the correct diagnosis when added to review of the conventional images. It is, however, important to consider cases where MRS and the DSS could have a negative impact. There was a reduction in the certainty of the correct diagnosis from review of MRS and/or DSS in only three cases. In two, the diagnoses were made correctly at all stages, with conventional imaging, with MRS and by the DSS, but the DSS gave a low probability. The other case was the myoblastic MB, which had MRS atypical of this tumour group. For the other two cases where the DSS was incorrect (both anaplastic EPs), the MRS and DSS did not have a negative impact on the certainty of diagnosis assigned by the radiologists. These cases had a low probability of diagnosis given by the DSS and were subsequently not given much weight in the radiologists' decision‐making. This indicates the likely importance of providing a measure of probability of the proposed diagnosis in the DSS.

This study shows the added value of MRS, over conventional MRI, in distinguishing between the three most common paediatric posterior fossa tumours. However, other tumour types do, albeit rarely, occur in this region of the brain in children. Building classifiers for these very rare tumours is a major challenge due to difficulties in collecting sufficient numbers of cases, but will emerge as further data are acquired.

Simply viewing MRS processed by the MR scanner software increased the radiologists' diagnostic accuracy and certainty of the correct diagnosis when added to conventional imaging. This reflects other paediatric studies in which adding MRS to conventional MRI significantly improved radiologists' diagnostic accuracy of brain tumours^23^, ^24^ and indicates that MRS can be a useful adjunct to MRI without sophisticated decision support software. The DSS gave both a comparison of the MRS with mean MRS from the three types of tumour and the results of a pattern recognition‐based classifier. We did not formally assess which of these components was most useful in aiding diagnosis, but as mean spectra of the tumour types were available from the literature during the visual interpretation of spectra, it is likely that the pattern classifier was more important. The radiologists were asked to provide comments as part of their assessment concerning which factors were most important in their final decision. However, such comments were general in nature and mostly stated whether the MRS/DSS was useful/not useful without specifying in detail how the MRS or DSS was interpreted. Overall, the pattern classifier gave a higher overall diagnostic accuracy than radiological review, even with the use of the DSS. Reliance on automated classification alone does, however, have significant potential pitfalls, particularly where the MRS is of poor quality. Of the three tumours misclassified by the DSS, one EP was correctly classified by the radiologists. On review of the radiologists’ comments, all three clinicians correctly classified the tumour based on location and appearance in the MR images. Making all imaging information available to the radiologist and providing appropriate clinical decision support is therefore recommended.

This study was performed using 1.5‐T MR scanners. This allowed a previously published classifier to be prospectively evaluated over a long enough period to acquire sufficient cases to evaluate it. However, many children with brain tumours now have their MRI performed on 3‐T scanners and there is emerging evidence that accuracy of classification using MRS is greater on these scanners.^9^ At the same time, the diagnosis using conventional MRI may also be greater at the higher field strength, making it difficult to predict the added value of MRS classifiers combined with clinical decision support in this situation. Further studies of the added value of MRS at 3 T should therefore be undertaken. In addition, the current study was performed at a single centre, and while this promotes conformity of acquisition, data‐handling robustness and ease of training radiologists in the study protocol, prospective studies of MRS classifiers and their added value for noninvasive diagnosis should also be undertaken in a multicentre setting. Having said this, it is encouraging that multicentre studies have shown the accuracy of MRS machine‐learning classifiers at both 1.5 and 3 T.^9^, ^17^

## 5 CONCLUSIONS

A MRS diagnostic classifier, previously evaluated retrospectively, was prospectively tested and yielded high accuracies for the three main childhood cerebellar tumours. Misclassified cases should be scrutinised by the clinical team for other atypical features before determining a management plan. Viewing the MRS increases both the diagnostic accuracy and the certainty assigned to the correct diagnosis compared with the conventional imaging alone. Additional improvements are made when a DSS based on the MRS classifier is also interpreted.

## CONFLICT OF INTEREST

None to declare.

## Data Availability

The data that support the findings of this study are available on request from the corresponding author. The data are not publicly available due to privacy or ethical restrictions.
